# vNARs as Neutralizing Intracellular Therapeutic Agents: Glioblastoma as a Target

**DOI:** 10.3390/antib13010025

**Published:** 2024-03-18

**Authors:** Alejandro Manzanares-Guzmán, Pavel H. Lugo-Fabres, Tanya A. Camacho-Villegas

**Affiliations:** 1 Unidad de Biotecnología Médica y Farmacéutica, Centro de Investigación y Asistencia en Tecnología y Diseño del Estado de Jalisco (CIATEJ), Guadalajara 44270, Mexico; almanzanares_al@ciatej.edu.mx; 2 Consejo Nacional de Humanidades, Ciencias y Tecnologías (CONAHCYT)—Unidad de Biotecnología Médica y Farmacéutica, Centro de Investigación y Asistencia en Tecnología y Diseño del Estado de Jalisco (CIATEJ), Guadalajara 44270, Mexico; plugo@ciatej.mx

**Keywords:** cancer immunotherapy, glioblastoma, molecular targeted therapy, variable new antigen receptors (vNARs), intrabodies, receptor tyrosine kinase

## Abstract

Glioblastoma is the most prevalent and fatal form of primary brain tumors. New targeted therapeutic strategies for this type of tumor are imperative given the dire prognosis for glioblastoma patients and the poor results of current multimodal therapy. Previously reported drawbacks of antibody-based therapeutics include the inability to translocate across the blood–brain barrier and reach intracellular targets due to their molecular weight. These disadvantages translate into poor target neutralization and cancer maintenance. Unlike conventional antibodies, vNARs can permeate tissues and recognize conformational or cryptic epitopes due to their stability, CDR3 amino acid sequence, and smaller molecular weight. Thus, vNARs represent a potential antibody format to use as intrabodies or soluble immunocarriers. This review comprehensively summarizes key intracellular pathways in glioblastoma cells that induce proliferation, progression, and cancer survival to determine a new potential targeted glioblastoma therapy based on previously reported vNARs. The results seek to support the next application of vNARs as single-domain antibody drug-conjugated therapies, which could overcome the disadvantages of conventional monoclonal antibodies and provide an innovative approach for glioblastoma treatment.

## 1. Introduction

Glioblastoma (GBM) is the most prevalent and fatal form of primary brain tumors, even under the standard of care treatment, which involves maximal safe surgical resection, radiotherapy, and chemotherapy. The overall survival (OS) rate was found to be only 5% among patients after 5 years of multimodal therapy. While antibody-based therapeutics are highly specific, they are usually limited to extracellular antigens. Consequently, monoclonal antibodies (mAbs) offer limited cancer cell membrane translocation, which makes it difficult to neutralize intracellular targets due to the molecular weight of mAbs (150 kDa). Unlike conventional antibodies, heavy chain single-domain vNARs have shown a higher capacity to infiltrate cells due to their smaller molecular weight (12–15 kDa). Thus, vNARs represent naturally occurring antibody-like molecules. One surprisingly overlooked application of single-domain vNARs is their application as intracellular delivery systems. Few studies have reported the intracellular delivery of heavy single domains (sdAbs) or their use as intracellular signaling cascade neutralizers. Therefore, vNARs represent an exciting unexplored field, as extensive research and further elucidation of the mechanisms involved in GBM pathogenesis may yield beneficial outcomes in GBM therapy. This review comprehensively summarizes the key intracellular pathways implicated in glioblastoma cell proliferation, progression, and survival as targets for glioblastoma therapy. Furthermore, we analyze the potential of previously reported vNARs as part of the next generation of vNAR drug-conjugated therapy to neutralize intracellular targets. Finally, we provide an update on the intrabody application of vNARs and an analysis of possible applications and future perspectives to advance the scientific debate and innovative approaches for vNARs.

## 2. Glioblastoma

GBM is the most prevalent and fatal form of primary brain tumors, accounting for ~50% of all gliomas [1]. Based on the WHO classification, GBM (grade IV) is characterized by tumors that steadily prompt mitotic activity, usually with necrosis and microvascular proliferation (or both) [2]. GBM is assumed to arise from neuroglial stem or progenitor cells through genetic alterations [3]. The incidence rate of GBM is 3.2 per 100,000 people, and approximately 17,000 new GBM cases are diagnosed yearly [4,5,6]. Despite the current multimodal-based standard of care, ~70% of GBM cases inevitably progress following one year of diagnosis, resulting in a clinical outcome that remains lethal for patients [3,7,8]. The average overall survival (OS) for GBM patients is 14.6 to 20.5 months [9,10,11,12,13], and the survival rate is <5% within the next 5 years after diagnosis [14]. In glioblastoma, mutations of the signaling pathways have been acknowledged, including anomalous stimulation of receptor tyrosine kinase (RTK) genes, phosphatidylinositol-3-OH kinase (PI3K), and p53, and retinoblastoma tumor deactivation of suppressor pathways [15]. These mutations lead to uncontrolled cell proliferation and decreased apoptosis, providing GBM tumor cells evasion mechanisms against cell-cycle checkpoints and apoptosis [16,17]. Distinctive genetic alterations such as overexpression of the epidermal growth factor receptor (EGFR), the lack of chromosome 10q, and phosphate and tensin homolog (PTEN) mutations have also been reported in GBM [17,18].

GBM remains incurable due to its distinctive molecular features, high recurrence after multimodal therapies, and unsatisfactory prognosis [1]. Subsequent chemotherapy and radiotherapy resistance in GBM are due to a population of self-renewing glioma stem cells (GSCs) [19,20]. Moreover, GBM cells invade neighboring healthy brain tissue, hampering the tumor’s maximal resection and neutralizing the effects of radiotherapy [1,21]. The presence of the blood–brain barrier (BBB) also represents an obstacle to better outcomes [1]. Given the inability of current multimodal therapies to improve GBM treatment in patients, new targeted therapeutic approaches are imperative. Such therapies could ameliorate undesirable adverse effects while increasing antitumor responses to achieve significant therapeutic outcomes.

## 3. vNARs as Potential Therapeutic Intracellular Single Domains for Glioblastoma

### 3.1. Conventional Antibody, Single-Domain VHH, and vNAR Features

**Figure 1:**
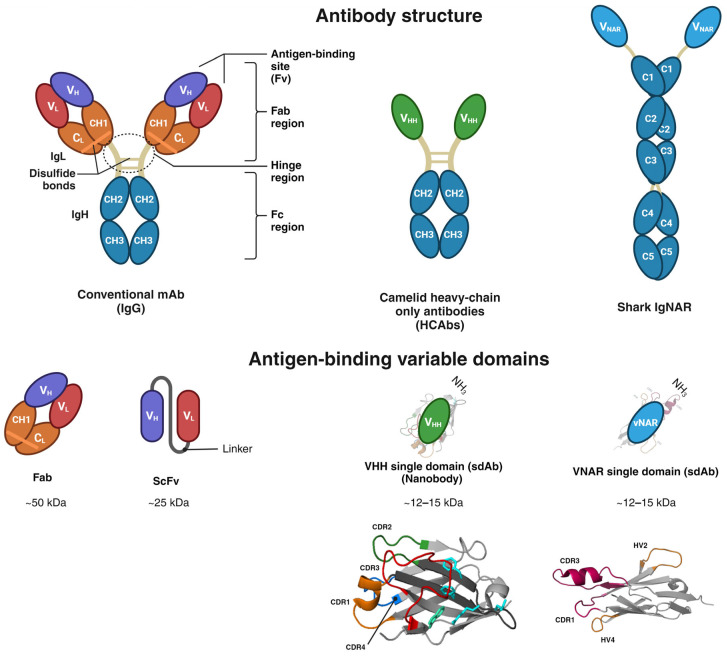
Structural domain comparison of conventional IgG, camelid heavy chain-only Igs, and shark IgNAR. IgG antibody structure comprises two light chains (IgL) and two heavy chains (IgH). The N-terminus holds variable domains within their corresponding heavy and light chains (VH and VL). The C-terminus encompasses constant domains (CH and CL). The fragment antigen-binding region (Fab region) contains a variable domain (V-domain) and a constant domain (C-domain) corresponding to two heavy chains and constant domains (CH2 and CH3) and an antigen-binding variable single domain (VHH). IgNAR forms a homodimeric structure comprised of two layers of five constant domains (C1 to C5) sandwiched as β-sheets joined through a hidden disulfide bond. The antigen-binding variable single domain (vNAR) at the N-terminus. The 3D structures of vNAR and VHH were adapted from [48,49], Copyright © 2021 Fernández-Quintero, Seidler, Quoika and Liedl, Copyright © 2017 Gonzalez-Sapienza, Rossotti and Tabares-da Rosa.

Antibody-based therapeutics can recognize and bind to extracellular cell surface receptors or soluble proteins. Once the antibodies bind to their antigen, signaling cascades are triggered, prompting cellular feedback, including cell proliferation, apoptosis, and nuclear factor activation. Despite the prominent specificity of antibodies, one of their major drawbacks is their inability to translocate across cancer cell membranes to reach intracellular targets, limiting them to extracellular antigens due to their size, hydrophilic structure, and endosomal entrapment. These factors translate into insufficient mAb translocation in the cytosol [22,23,24,25]. Therefore, the efficacy of mAbs is restrained by their significant molecular weight (~150 kDa) and intrinsic physical complexity, limiting their intracellular delivery and paratope accession to antigens with cryptic epitopes and ultimately yielding poor binding affinities [26,27]. The IgG antibody comprises a pair of light chains and a pair of heavy chains connected by a disulfide bond, presenting a quaternary Y-shaped structure [28,29]. The N-terminus holds the variable domains within the heavy and light chains (VH and VL). The C-terminus encompasses constant domains (CH and CL). The fragment antigen-binding region (Fab region) contains a variable domain (V-domain) and a constant domain (C-domain) correspondingly found in both the L chain and H chain of the antibody. The fragment crystallizable region (Fc) is located at the base antibody (Figure 1) [29]. In 1993, a heavy-chain antibody was also discovered in camelids (Figure 1) [30].

Cartilaginous fish, including skates, sharks, sawfish, and rays, appeared nearly 450 million years ago and share ancestral features with other vertebrates [29,31]. Shark Ig antibodies were identified in Ginglymostoma cirratum (nurse shark) in 1995 and fall into the following three classes: IgM, the earliest class in vertebrate evolution [32]; IgW, orthologous to the mammalian IgD isotype [33]; and IgNAR, found at a serum concentration of 0.1–1.0 mg/mL, representing the critical antibodies within the adaptive immune systems of sharks [29,34,35]. IgNAR has a heavy chain-only homodimer, unlike standard antibodies, which lack the typical light chain association. Thus, antigen binding is performed only by a dyad of autonomous variable single domains and the shark variable domain of the new antigen receptor (vNAR), which are highly soluble. Additionally, each vNAR possesses a molecular weight of ~12–15 kDa, representing the smallest naturally occurring antibody-like molecule (Figure 1) [29,36]. The secreted homodimer IgNAR comprises two layers of five constant domains (C1–C5) sandwiched with β-sheets, joined by a hidden disulfide bond, and the antigen-binding variable single domain (vNAR) at the N-terminus. vNAR is connected to the constant domains through a flexible hinge region (Figure 1) [27,29,32,37]. The vNAR single domain is acknowledged as a potential tool for diagnosis and immunotherapy due to its small molecular weight, high stability, solubility, and targeting capability toward cryptic antigen sites and enzyme active sites [29,38]. The Ig superfamily encompasses vNAR, and its structure is characterized by a β-sandwich fold incorporating eight β-strands produced by framework 2 (FR2)-CDR2 region removal, unlike the ten β-strands found in mammalian V domains. In addition, vNARs contain only two regions of high variability, CDR1 and CDR3, unlike the mammalian variable region with three CDRs [27,29]. CDR3 is indeed the most divergent vNAR region. Upon antigen exposure, a somatic mutation occurs in vNAR, as revealed in CDR1, a truncated CDR2 site, as well as a loop of TCR–HV4. These mutation-prone regions are termed HV2 and HV4 (Figure 1) [29,39]. Likewise, vNARs demonstrated a higher antigen-binding affinity through their four antigenic-binding loops (CDR1, CDR3, HV2, and HV4), in contrast to traditional antibodies, which are composed of six loops throughout two chains [29,40,41]. The vNAR single-domain binding affinities through CDR3 have been reported in the nanomolar range toward an antigen [40,42], and the lowest binding affinity was reported at the picomolar range for the recognition of human serum albumin (anti-HSA vNAR) [41]. Therefore, the molecular structure of vNAR represents a fascinating tool for developing scientific research, especially in diagnostic and therapeutic approaches.

Compared to camelid variable heavy domain of heavy chain (VHH), vNARs are smaller; they only have CDR1 and CDR3, and they lack a CDR2 region (Figure 1). Instead, vNARs have two hypervariable regions (HV2 and HV4) [43]. HV2 and HV4 have demonstrated a greater frequency of somatic mutations, indicating their potential involvement in antigen recognition [42]. The variable region of IgNAR determines the specificity of the antibody. Therefore, vNAR types are defined in terms of their cysteine number, CDR3 length, and amino acid variability [43]. Due to the absence of CDR2 in vNAR, sequence diversity is compensated in the CDR3 region. vNARs possess very structurally complex and long CDR3s, which exhibit a high degree of variability to counterbalance the reduced size of variable regions in IgNARs [43,44]. Therefore, CDR3 is more variable in sequence, length, and conformation, giving it a crucial role in antigen identification [43]. The natural absence of CDR2 in vNAR exacerbates the requirements for CDR1 and CDR3 to provide specific and high-affinity binding to potential antigens [21,45]. The vNAR single domain with four antigen-binding loops over a single chain has been shown to bind antigens with relatively higher affinity than conventional antibodies containing six CDR loops across two chains [40,43]. Evolutionary mechanisms in VHH have been adapted to compensate for repertoire diversity due to a lack of the VL domain, including extended CDR1, longer CDR3, and the involvement of FR2 in antigen binding and modeling the CDR3 loop, which comprise the role of the CDR4 (residues 76–80) loop in antigen binding and extensive somatic hypermutation. As a result, there may be limitations to the extent of manipulation and engineering that can be tolerated by VHH [46,47]. A comparison of VHH and vNAR is summarized in Table 1. Recently, three companies globally have been involved in the research and development of shark antibody drugs: Ossianix, Elasmogen, and AdAlta [43].

**Table 1:** Comparison between VHH and vNAR.

	VHH	vNAR	Refs.
**Similarities**	(1) Heavy chain-only single domains. (2) Promising candidates for biomedical development. (3) Small size, high specificity for analogous antigens, and high physiochemical stability. (4) A wide range of loop lengths and structures. (5) Access to cryptic epitopes and catalytic clefts of enzymes. (6) Short half-life in blood circulation. (7) Humanization could be necessary to limit potential immunogenicity. (8) A variety of recombinant expression systems available.		[25,50,51,52,53,54]
**Differences**	(1) Isolated from camelids (camel, llama, alpaca, and dromedary). (2) CDR2 site present. No HV2 nor HV4 regions. (3) Three antigenic-binding sites (CDR1, CDR2, and CDR3).	(1) Isolated from Cartilaginous fish (skates, sharks, sawfish, and rays). (2) Truncated CDR2 site and replaced by short HV2. (3) Presence of an HV4 region. (4) Smallest antigen-binding domain related to their eight β-strands. (5) Four antigenic-binding sites (CDR1, CDR3, HV2, and HV4).	[16,21,29,31,39,40,41,44]
**Advantages**	(1) High homology to human VH scaffolds (>80%). (2) Ability to target antigenic epitopes at locations difficult to access by mAbs (such as G protein-coupled receptors and ion channels). (3) VHHs are suitable for such applications due to their small size, target specificity, and long CDR3 loops, bypassing numerous drawbacks related to small-molecule synthetic drugs such as specificity and a lower risk of off-target toxicity.	(1) vNAR encodes an unusually long (20–25 amino acids) and structurally complex CDR3. (2) Presence of canonical cysteines and extra cysteines along the CDR3, providing additional disulfide bonds that impact the structural diversity. (3) The extensive CDR3 of vNARs is a considerable advantage, resulting in a long loop that favors access to cryptic epitopes of target antigens (such as pockets or grooves) and high tissue penetration.	[38,43,44,46,47,55,56,57,58,59,60,61,62,63,64,65]
**Disadvantages**	(1) Each amino acid (depending on its position) may have direct and indirect effects on the molecule’s stability and structural integrity, as well as on antigen-binding affinity and specificity. (2) VHHs have a low propensity to bind small molecules, likely owing to their dominant convex surface topology compared to the flat or concave topologies found on conventional antibody fragments (e.g., scFv, Fab).	(1) Low homology (25–30%) and identity were found between vNARs and the human VH domain. (2) Low recombinant expression depends on a selected heterologous expression system. (3) Concerns related to the safety of pharmaceutical administration in humans.	[39,43,46,47,65]

VHHs: variable domain of the heavy chain of heavy-chain antibodies; vNARs: variable new antigen receptors; CDR: complementarity-determining region; HV: hypervariable region.

### 3.2. Single-Domain VHH and vNAR Intrabodies for Cancer

Currently, there are examples of the effectiveness of vNAR. The potential of vNARs as diagnostic and therapeutic tools continues to prompt intensive research into new targets [55,66,67,68]. Nevertheless, few studies have reported on both intracellular antibody delivery and downstream cellular responses. Studies regarding vNARs as intracellular therapeutic agents are even more limited. In contrast to conventional antibodies, vNAR and VHH have shown higher stability and solubility and a potentially greater capacity to infiltrate cells because their smaller molecular weight (~12–15 kDa) grants them the ability to permeate tissues and target cryptic epitopes within cells [48,67]. Moreover, vNARs can be employed for targeted therapy due to their specific epitope recognition tailored by phage-display technology and semisynthetic or synthetic approaches.

An intrabody is an antibody fragment designed to be intracellularly expressed [69]. Intrabodies can target endogenous or ectopic intracellular antigens within living cells. Intrabodies have been applied as chromobodies for live cell microscopy and biosensors to sort out the intricate cellular signaling pathways. Moreover, specific protein knockouts or the modulation of intracellular targets can be achieved by specific nanobodies, granting them the potential to act as prospective therapeutics [70]. The intrabody strategy has been employed effectively for oncologic targets such as Epidermal Growth Factor Receptor (EGFR) and Vascular Endothelial Growth Factor Receptor 2 (VEGF-R2) [25,71,72,73]. Single-chain antibody fragments (scFv) are the most prevalent format for intrabodies due to the simplicity of their relative expression and intracellular stability compared to full-length IgG antibodies [25,71,73]. Nevertheless, the ability of scFvs to endure the harsh pH and proteolytic activity within the lysosome represents an obstacle. Thus, a more robust biological scaffold is needed. Single domains such as VHH and vNAR represent a promising alternative due to their unique structural features and stability [70,71]. Diverse receptors and transcription factors can be targeted by nanobodies, hindering dysregulated signaling pathways that lead to the unrestrained growth and migration of cancer cells [70]. A summary of VHH and vNAR intrabodies reported for cancer is presented in Table 2.

The classical intrabody method employs only the sequence of the nanobody (VHH) or vNAR, which can be coupled to various fusion partners via standard molecular biology technologies. The subsequent transfer of the genetic information of DNA or mRNA into living cells through different transfection or transformation procedures such as electroporation, lipofection, viral-vector-based delivery, or the transfer of nanoparticles leads to nanobody expression by the cellular transcription/translation machinery [50,70,74,75,76,77]. Furthermore, the intrabody can be precisely directed to distinct compartments within the cell by incorporating ER localization as well as mitochondrial, nuclear, and/or retention sequences.

Previous studies have shown that recombinant vNAR is stable when exposed to harsh environments such as extreme pH and proteinase hydrolytic cleavage in mouse gastric scraping (pH = 5), as well as intestinal samples (a proteinase-rich environment), with no evident signs of degradation after 1 h of incubation [67,68]. Additionally, vNAR was incubated for 3 h at high temperatures (85–97 °C) and still preserved the binding activity [40].

**Table 2:** Summary of VHH and vNAR intrabodies against cancer.

Intrabody Name	SdAb	Target	Cancer	Approach	Results	Ref.
vNAR clone 3 and vNAR clone 5	vNAR	Cysteine protease Cathepsin S (CTSS) (ProCTSSC^C25S^)	Colon cancer, Burkitt’s lymphoma, and glioblastoma	CTSS accelerates tumor progression via angiogenesis, contributing to tumor cell invasion and migration through the degradation of the extracellular matrix (ECM).	Novel mechanistic approach to hamper CTSS deleterious activity. vNARs demonstrated inhibition of intracellular CTSS. vNARs prevented the activation of the CTSS proenzyme. Treatment with vNAR clones attenuated the invasive nature of the 251-cell line across an ECM mimetic matrix assay.	[71]
Nb64	VHH	α-actinin-4 (ACTN4)	Prostate cancer	PI3K/AKT-driven signaling pathways interfere by targeting an actin-binding protein, α-actinin-4 (ACTN4).	Intracellular expression of Nb64 hampered proliferation, migration, and invasion in prostate cancer cell lines.	[78]
VHH212	VHH	Transcription factor Hypoxia induced factor 1α (HIF-1α)	Pancreatic ductal adenocarcinoma (PDAC)	HIF-1α has a critical role in cell tumor progression and progression under hypoxic conditions.	VHH212 neutralizes the HIF-1α function in PDAC tumors associated with gemcitabine treatment.	[79]
VH18, VHH35 and VH36	VHH	EGFR	Lung adenocarcinoma	Inhibit EGFR-TK intracellular signaling.	Nbs were shown to be advantageous tools for the study of downstream TK signaling. Possible candidates for clinical application, especially VH36, might disrupt EGFR dimerization, leading to the inhibition of intracellular signaling.	[80]
SBT-100	VHH	STAT3	Breast cancer	Inhibition of constitutive expression and activation of STAT3, impaired in 70–80% of human malignancies.	SBT-100 selectively target STAT3, impairing signal transduction owing to loss of function of the phosphorylated STAT3. In a human breast cancer xenograft model with MDA-MB-231 cells, tumor growth decreased after intraperitoneal treatment with SBT-100. Internalization mechanism is not specified. SBT-100 is hypothesized to cross the cell membrane.	[81]

**STAT3: signal transducer and activator of transcription 3.**

### 3.3. The Influence of Epidermal Growth Factor Receptor Amplification and Epidermal Growth Factor Receptor Variant III in Glioblastoma

**Figure 2:**
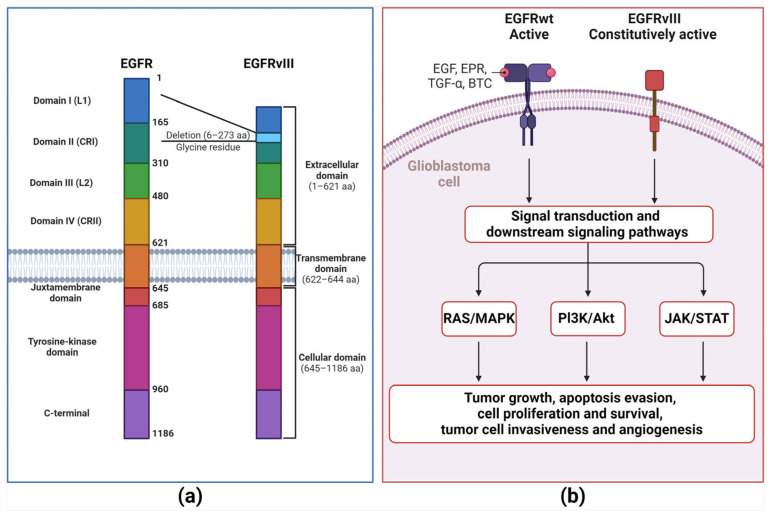
EGFR wild type and EGFRvIII mutated comparison. (**a**) The epidermal growth factor receptor (EGFR) comprises three domains: an extracellular domain, a transmembrane domain (hydrophobic), and an intracellular domain (distinctive of EGFRs from the TKI family and highly conserved). The extracellular domain comprises four smaller domains (DI-DIV). DI and DII are essential to ligand binding. EGFRvIII was obtained because the 801 bp in-frame deletion of 2–7 exons of the EGFR gene. (**b**) The extracellular domain interacts with numerous ligands, including epidermal growth factor (EGF) protein, EGFR-like growth factors, epiregulin (EPR), transforming growth factor-alpha (TGF-α), and betacellulin (BTC). EGFR is activated by ligand binding followed by dimerization, which provokes a conformational shift that further supports EGFR intracellular triggering by specific phosphorylated tyrosine residues (Y845, Y992, Y1068, Y1086, and Y1173) at the carboxyl-terminal domain, followed by activation of a complex program of downstream intracellular signals within the cytoplasm and nucleus, including RAS-MAPK, phosphatidylinositol-3-kinase (PI3K)/Akt and signal transducer and activator of transcription 3 (STAT3) pathways. These downstream signaling cascades prompt cell proliferation, loss of differentiation, invasion, angiogenesis, and inhibition of apoptosis. EGFRVIII maintains intact transmembrane and intracellular kinase domains, granting EGFRvIII independence of ligand binding to support further growth signaling in GBM cells and malignancy.

Epidermal growth factor receptor (EGFR, also known as HER1/ErbB1) within the tyrosine kinase family is a transmembrane glycoprotein of 170 kDa composed of 1186 amino acid residues as a single-polypeptide chain, corresponding to HER1-HER4 receptor members [51]. EGFR comprises three domains: an extracellular domain, a transmembrane domain (hydrophobic), and an intracellular domain (distinctive from EGFRs of the TKI family and highly conserved). The extracellular domain, which is composed of four smaller domains (DI-DIV), participates in ligand binding through its DI and DIII domains [51,82,83,84]. Thus, this domain interacts with ligands, including epidermal growth factor (EGF) protein, EGFR-like growth factors, epiregulin (EPR), transforming growth factor-alpha (TGF-α), and betacellulin (BTC) [51]. EGFR is triggered by the binding of the matching ligand, followed by dimerization and autophosphorylation of the intracellular domain, which elicits downstream signaling cascades. These cascades elicit downstream signaling cascades, including the pathways of RAS/MAPK, phosphatidylinositol-3-kinase (PI3K)/Akt, and signal transducer and activator of transcription 3 (STAT3) (Figure 2) [51,83,84]. EGFR is usually expressed in healthy cells and regulates cell proliferation. Moreover, EGFR is rigorously controlled by tissue homeostasis, which relies on cell proliferation. EGFR overexpression is correlated with poor clinical outcomes in solid tumors of human cancers, including breast, head, and neck cancers [51].

In GBM, almost 50% of the EGFR gene is amplified, yielding a distinctive tumor cell-specific variant termed EGFRvIII (de2-7EGFR/ΔEGFR). EGFRvIII is the most prevalent EGFR mutation in GBM, accounting for 25–33% of all GBM cases and is found only in malignant cells [85,86,87]. EGFRvIII develops from the 801 bp in-frame deletion of exons 2–7 of the EGFR gene [86], obliterating the extracellular domain with the elimination of 267 amino acids and crafting a new glycine residue among exons 1–8 [88]. However, EGFRVIII maintains the transmembrane and intracellular kinase domains intact, allowing ligand binding to independently engage in further growth signaling in GBM cells and malignancy (Figure 2) [86,89]. Moreover, EGFRvIII can be triggered through various intracellular proteins, including Src family kinases (Y845 and Y1101 sites). This intracellular protein kinase activation protects EGFR-positive cancer cells against EGFR inhibitors targeting the ectodomain [90,91]. Hence, EGFRvIII represents a promising aim for targeted therapy strategies.

### 3.4. Therapeutic Tyrosine Kinase Inhibitors and Anti-EGFR Monoclonal Antibodies in Glioblastoma

In cancer, passive immunotherapy relies on the administration of monoclonal antibodies (mAbs), which represent relevant therapeutic agents because of their capacity to combat cancer without requiring an active role in the host’s immune system [92]. In this way, mAbs recognize their corresponding cell surface antigens and lead to targeted apoptosis via antibody-dependent cellular cytotoxicity (ADCC) or complement-mediated cytotoxicity (CDC) [93]. Tumor-associated antigens (TAAs) encompass molecules found in normal cells. However, TAAs are overexpressed in malignant cells. Neoantigens are tumor-specific antigens (TSAs) that develop because of mutations in the malignant cell genome [94]. To diminish detrimental effects in healthy tissues, compelling mAb-targeted therapies involve the stable tumor-specific antigen cell surface expression of at least 1 × 10^5^ molecules per tumor cell [92,95].

**Figure 3:**
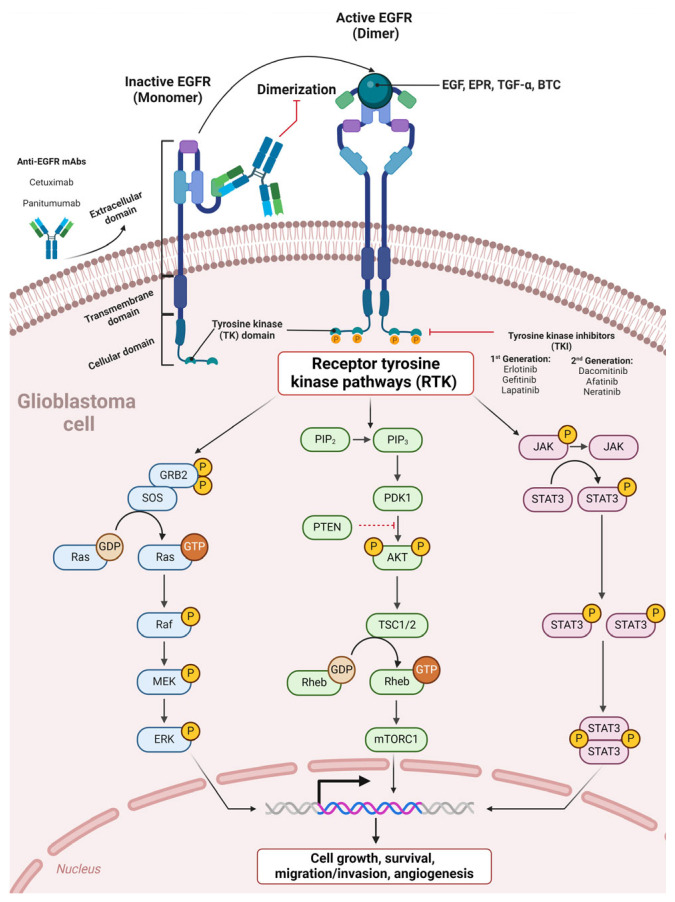
Therapeutic tyrosine kinase inhibitors and anti-EGFR monoclonal antibodies in glioblastoma anti-EGFR mAbs bind the EGFR extracellular domain, preventing EGFR dimerization and subsequent activation. Tyrosine kinase inhibitors (TKIs) target the intracellular TK domain, blocking subsequent receptor tyrosine kinase pathways (RTKs).

Several approaches impede expression and target EGFR through tyrosine kinase inhibitors (TKIs) and monoclonal antibodies. TKIs, including erlotinib, gefitinib, and lapatinib, target the intracellular TK domain, impeding proliferation signaling. Anti-EGFR mAbs, including cetuximab, hinder EGFR dimerization by targeting the EGFR extracellular domain (Figure 3). Nevertheless, despite previously reported outcomes, anti-EGFR agents showed discouraging results in clinical trials [51,96]. In GBM, EGFR inhibitors presented a difference between the degrees of signaling inhibition and clinical efficacy [97]. First-generation EGFR inhibitors demonstrated limited effects on EGFR signaling due to variable degrees of inhibition [98]. On the other hand, second-generation EGFR inhibitors (neratinib, dacomitinib, and afatinib) have yielded promising outcomes [97]. In the case of afatinib for GBM, a phase I/randomized phase II study demonstrated a greater progression-free survival (PFS) than that among untreated patients. This study included treatment with and without temozolomide (TMZ) in patients with first or second confirmed GBM recurrence harboring tumors with high EGFR amplification levels, as well as the immunoreactivity of EGFRvIII or absence of PTEN [97,99]. Cetuximab was found to be tolerable in phase II clinical trials for recurrent GBM but demonstrated unsatisfactory efficacy associated with low BBB diffusion, which impedes the mAbs from reaching the tumor [97,100].

### 3.5. Single-Domain VHH and vNAR Cellular Internalization in GBM

TfR1-mediated (transferrin receptor 1) transcytosis is a promising technique for increasing the uptake of protein therapeutics in the brain. A single-domain shark antibody vNAR fragment (called TXB2) targeted transferrin receptor 1 (TfR1) with equal affinity to human and murine TfR1. TXB2 was employed to deliver the protein cargo to the brain [29,101]. TXB2-hFc fusion showed significant brain uptake in vivo through TfR1 transport mechanisms. Thus, TXB2 was found to be a brain-selective, species-cross-reactive, and high-affinity vNAR antibody to TfR1 capable of crossing the BBB rapidly and with suitable safety and a pharmacokinetic profile. TXB2 can be easily adapted as a carrier for a broad diversity of biotherapeutics, from the blood to the brain [101]. TXB2’s fusion with Bapineuzumab, an anti-amyloid targeting Aβ, demonstrated that targeted TfR1-mediated transcytosis for the brain delivery of an IgG antibody was indeed accomplished via fusion to the vNAR (TXB2) immunocarrier. Brain concentrations of Bapi-TXB2 were threefold higher than those in Bapineuzumab. In transgenic mice overexpressing human Aβ, the brain-to-blood concentration ratio increased over time due to interactions with intracerebral Aβ deposits. This threefold difference between Bapi-TXB2 and Bapineuzumab was observed for up to 6 days after the injection [101]. Moreover, several patents of Ossianix have been approved for vNARs capable of crossing the BBB [102,103,104,105]. This result demonstrated the capacity of vNAR to cross the BBB using an immunocarrier approach.

Cellular uptake is one of the most critical processes regulating the biological activity of molecules and is determined by interactions between the molecule and the plasma membrane. Receptor-mediated endocytosis (RME) is a vesicular transport event that cells use to initiate the endocytosis of activated cell surface receptors. This event occurs through the inward development of plasma membrane vesicles containing receptors with sites specific to the new internalized proteins [106]. Previous studies have exploited this mechanism for VHH and vNAR to target specific cell membrane receptors in GBM.

An anti-EGFRvIII nanobody (EG2-Cys) conjugated with a near-infrared quantum dot (Qd800) showed significant internalization in U87MG EGFRvIII-expressing cells in vitro compared to that in EG2-hFc conjugated with Qd800 or unconjugated EG2-hFc [107]. In an orthotopic glioblastoma mice model, EG2-Cys also demonstrated an improvement in the contrast of near-infrared imaging of the tumors. The targeting ability of EG2-Cys towards EGFRvIII demonstrated that sdAbs conjugated with Qd800 can provide specific detection in vitro and in vivo in EGFRvIII-expressing cells [107].

Photodynamic therapy (PDT) is a minimally invasive treatment modality that employs near-infrared light to activate a photosensitizer capable of eliminating cancer cells locally. However, one of the main aspects restricting PDT use in the clinic is the poor selectivity and hydrophobicity of the photosensitizer [108]. Photosensitizers have been successfully conjugated with VHH targeting cell membrane receptors to overcome non-selective delivery, leading to RME and internalization within the GBM cells. For example, the neutralization of the human cytomegalovirus (HCMV) chemokine receptor US28 by VHH is called VUN100. VUN100 inhibits constitutive US28 signaling and partially hampers US28-enhanced tumor growth in vitro and in vivo [109,110]. VUN100 was conjugated with the water-soluble photosensitizer IRDye700DX. This approach was intended to eliminate US28-expressing glioblastoma cells by exploiting VUN100-targeted PDT. The results demonstrated selective obliteration of US28-expressing glioblastoma cells in 2D cultures and 3D spheroids [109]. Anti-IL-13Rα2 vNARs (13R_VNAR_102 and 13R_VNAR_106) for GBM have been reported. These vNARs demonstrated a robust inhibitory ability on the growth and migration of highly expressed IL-13Rα2 cells [111]. These antigen receptors will be further detailed in Section 3.6.

Zottel et al., validated four VHHs (Nb79, Nb225, Nb179, and Nb314) targeting intracellular targets in GBM, including vimentin (VIM), mitochondrial translation elongation factor (EF-TU) (TUMF), nucleosome assembly protein 1-like 1 (NAP1L1), and dihydropyrimidinase-related 2 (DPYSL2) protein, respectively. Consecutive treatment with Nb79 (anti-VIM) and Nb225 (anti-TUFM) significantly decreased GBM cell survival (U87MG, U251MG, NCH644, and NCH421k), which was found to be the most effective for these GBM cell lines. However, no experimental information was available on the internalization mechanisms of these nanobodies into the cell membrane [112]. Although these observations are intriguing, a more detailed membrane translocation study would be advantageous. A summary of these single-domain antibodies against GBM and related details are provided in Table 3. A more detailed description of VHH and vNAR in the potential delivery and treatment of other CNS diseases was covered in recent reviews [113,114].

### 3.6. Inhibitory Effects Induced by the vNAR-Targeted Blocking of IL-13Rα2 on the GBM Cell Surface

IL-13Rα2 is a monomeric, high-affinity interleukin-13 (IL-13) receptor overexpressed in ~78% of GBM. This receptor is expressed at minimal levels or is absent in normal brain tissues and controls receptor-mediated endocytosis after binding to IL-13 [115,116,117]. IL-13Rα1 is a low-affinity IL-13 receptor. Upon IL-13 binding, IL13Rα1 forms a high-affinity heterodimer with IL4Rα, which mediates signal transduction via IL-13 receptors and IL-4 receptors [115].

**Figure 4:**
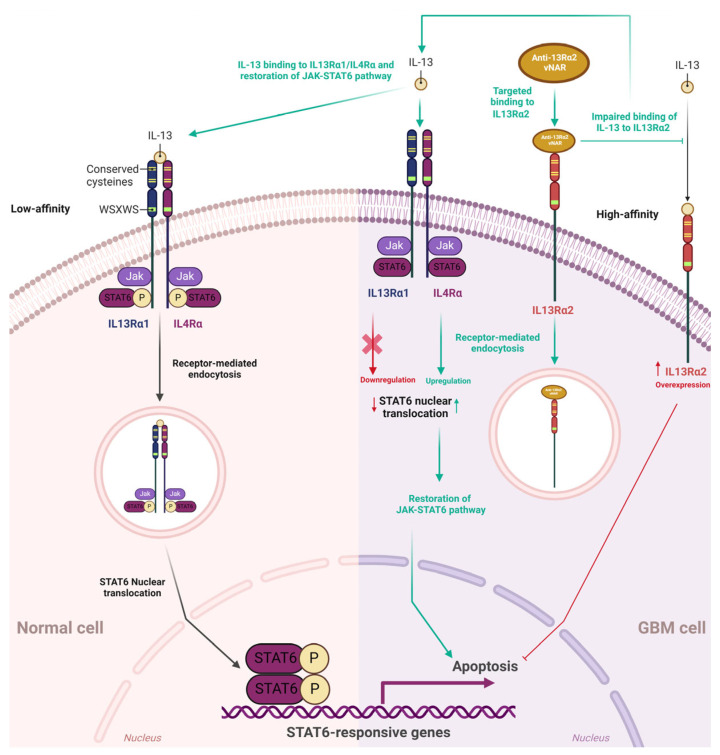
IL13Rα1/IL4Rα JAK-STAT6 pathway, IL13Rα2, and single-domain vNAR coupled to IL13Rα2. In normal cells, IL-13 binds with low affinity to IL13Rα1, and then IL13Rα1 forms the IL13Rα1/IL4Rα heterodimer with IL4Rα. IL13Rα1/IL-4Rα binding to the IL-13 cytokine stimulates STAT-6 intracellular signaling, resulting in receptor-mediated endocytosis (RME) and STAT6 translocation to the nucleus. IL-13Rα2 is a monomeric, high-affinity interleukin-13 (IL-13) receptor overexpressed in ~78% of GBM. IL-13Rα2 is absent or expressed at minimal levels in normal brain tissues. Anti-IL13Rα2 vNAR binds to IL13Rα2 on the GBM cell surface, then IL13Rα2 undergoes receptor-mediated endocytosis and hampers IL-13 binding to IL13Rα2. Consequently, IL-13 can normally bind to IL13Rα1/IL4Rα, further initiating the JAK-STAT6 pathway; this leads to the restoration of this signaling pathway that inhibits the abnormal unlimited proliferation of GBM cells and finally culminates in the apoptosis of these cells. Green dashed and green solid lines depict indirect and direct effects of IL13Rα2 impairment by targeted binding of vNAR, respectively. Red lines and the red cross depict IL13Rα2 inhibition of the JAK-STAT6 signaling pathway through IL-13 binding.

IL-4 and IL-13 phosphorylate different JAK kinases in solid tumor cells via IL-4Rα and IL13Rα1. However, these phosphorylation processes activate the same STAT6 protein. IL13Rα1/IL-4Rα binding to IL-13 prompts the activation of STAT-6 signaling, resulting in translocation to the nucleus [118]. Conversely, the IL13Rα2 chain does not signal through the STAT6 pathway [115] (Figure 4). IL-13 can signal via IL13Rα2 in a STAT6-independent manner with AP-1, prompting TGF-β1 promoter activation and resulting in inflammation and fibrosis in animals [119].

Qin et al., obtained anti-IL13Rα2 vNARs from an immune library of *Chiloscyllium plagiosum* [111]. After rigorous analysis and validation with multiomics, anti-IL13Rα2 vNARs were effectively expressed in the *Escherichia coli* prokaryotic expression system. Recombinant 13R_VNAR_102 and VNAR_106 were evaluated at various concentrations (1, 5, and 50 μg/mL) to assess their inhibitory effects in A172 cells. Cell survival tests were determined using CKK-8 and compared to a positive control of bevacizumab (BVZ) for 24, 48, and 72 h. The inhibition effects were positively correlated with time and dosage. There were comparable effects between 13R_VNAR_102 (1 μg/mL) and BVZ. When concentrations of 13R_VNAR_102 were increased to 10 and 50 μg/mL, significantly improved inhibitory effects were observed compared to those in the BVZ group [111]. Similar results were found in 13R_VNAR_106 (10 μg/mL) and BVZ and 13R_VNAR_106 (50 μg/mL) versus BVZ.

To further confirm the specificity and inhibitory effects of vNARs, IL13Rα2 was silenced via siRNA interference in A172 cells and assessed using different vNAR concentrations (0, 1, 10, and 50 μg/mL). Unrelated siRNA-treated A172 cells were set as the control group [111]. CCK-8 tests were performed 24, 48, and 72 h after vNAR incubation. Upon silencing IL13Rα2 in the A172 cells, incubation with vNAR showed limited growth-inhibitory effects compared to those in the control group. In another assay, the inhibition of the screened vNAR was recovered after the cells were re-expressed with IL13R2α2 after transfection with pcDNA3.1(+)-IL13Rα2. The assessed vNARs demonstrated significant inhibitory potential on the A172 cells with high expression of IL13R2α2. These findings suggest that these vNARs could effectively inhibit cell growth by binding to IL13R2α2 on the A172 cell surface [111]. According to the authors, these findings could be attributed to the binding of these recombinant vNARs to the IL13R2α2 receptor on the cell surface. Further IL13Rα2-mediated internalization hampers IL-13 binding to IL13Rα2. Thus, IL-13 can normally bind to IL13Rα1/IL4Rα, initiating the subsequent JAK-STAT pathway that inhibits unlimited proliferation and induces apoptosis of tumor cells [111,120] (Figure 4). In a wound healing assay, recombinant IL13Rα2 vNAR demonstrated significant inhibition of A172 cells in a dose-dependent manner. Furthermore, the JAK-STAT6 signaling pathway was negatively regulated by IL13Rα2 [119]. Intriguingly, the inhibition of IL13Rα2 by targeted vNAR or siRNA significantly activated STAT6 in A172 cells, which could explain the subsequent inhibitory effects (or apoptosis) in these GBM cells. Overall, these findings provide an experimental basis for developing targeted inhibition of IL13Rα2, which could represent a beneficial therapeutic approach and highlight the relevance of developing novel vNARs capable of recognizing tumor-associated targets that selectively inhibit the malignant progression of glioma.

## 4. vNARs as Potential Neutralizers of Intracellular Signaling Pathways

In the previous section, we considered single domains with their corresponding advantageous features as remarkable tools for intracellular delivery. As previously stated, several examples of single domains as intrabodies have been intracellularly expressed to inhibit crucial signaling pathways, which may be beneficial in hampering the cell growth, proliferation, and migration of cancer cells. These single domains are summarized in Table 2. Another remarkable internalization mechanism that single domains can exploit is receptor-mediated endocytosis (RME), which can be accomplished through the targeted binding of single domains to the receptor. Specific examples of sdAbs directed to GBM tumor-associated receptors are also summarized in Table 3. These findings suggest that developing intracellular sdAbs that selectively recognize tumor-associated targets and inhibit relevant signaling pathways may provide a more advantageous therapeutical approach to prevent the malignant progression of tumors. Next, we describe the receptors and intracellular proteins involved in the relevant GBM intracellular signaling pathways. We also further explore the potential of employing previously described vNARs that precisely bind to these targets but have yet to be considered in GBM. Therefore, vNARs may be employed for the targeted inhibition of relevant intracellular signaling pathways involved in glioblastoma pathogenesis via intracellular expression as intrabodies or to impede ligand binding to the cell membrane receptors, as well as subsequent downstream signaling.

### 4.1. vNAR Domain for the Allosteric Inhibition of Aurora-A Kinase

**Figure 5:**
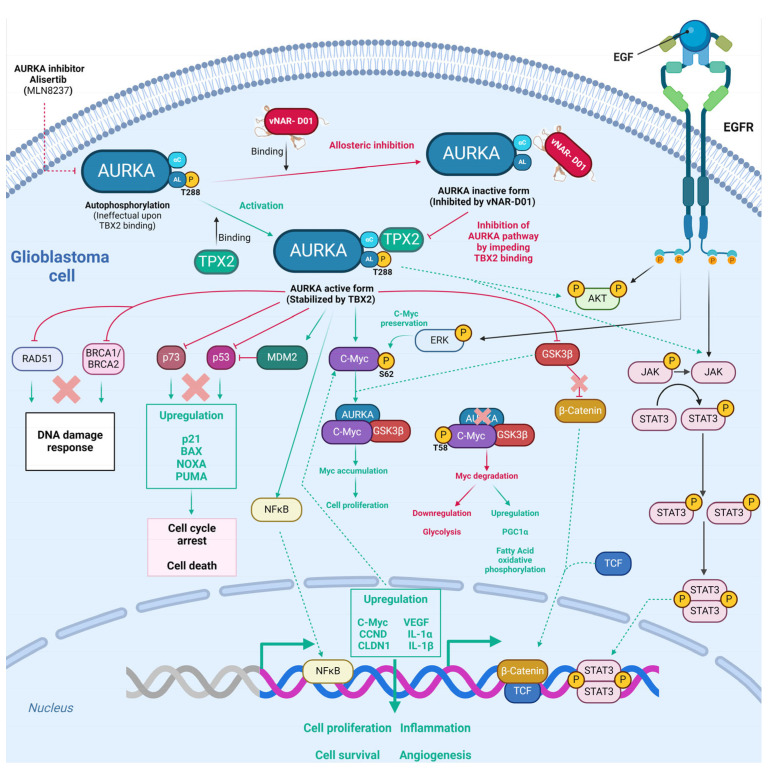
Potential allosteric inhibition of Aurora-A kinase by the vNAR-D01 intrabody. Aurora-A is a Ser/Thr protein kinase primarily involved in cell division and has been proven relevant for proliferation in glioblastoma. Moreover, Aurora-A induces tumorigenesis via the decontrolled regulation of RAD51 [128]. BCRA1, catenin, p73, MDM2, NFκB, cMyc, ERK, AKT, and JAK/STAT pathways [130], followed by downstream upregulation of several targets that prompt GBM cell proliferation, survival, inflammation, and angiogenesis. AURKA autophosphorylation is inefficient and relies on TPX2 binding. vNAR-D01 binding superposes to recognition sites of TPX2, further impeding AURKA kinase activity via an allosteric mechanism. Because the vNAR-D01 binding superposes to recognition sites of TPX2, the former impedes AURKA kinase activity via an allosteric mechanism. Green solid lines depict direct regulation, and green dashed lines depict indirect regulation. Red lines depict inhibition as well as red crosses. The vNAR-D01 3D structure was adapted for [122].

Aurora-A kinase (AURKA) is a Ser/Thr protein kinase involved in cell division that contributes to the proliferation and growth of solid tumors, including glioblastoma [121,122,123]. The AURKA-mediated phosphorylation of downstream substrates such as n-Myc/c-Myc transcription factors maintains cell cycle regulation, which further sustains cancer progression [122,123,124,125]. c-Myc (MYC) is a transcription factor (oncogenic) that eases tumor growth partially via metabolic regulation. Since c-Myc has a short half-life, its preservation relies on Serine 62 (S62) ERK-mediated phosphorylation and degradation on Threonine 58 (T58) GSK3β-mediated phosphorylation [123,126]. Moreover, AURKA triggers tumorigenesis via decontrolled regulation of the BRCA1, p53, NFκB, and mTOR pathways (Figure 5) [127,128,129,130,131,132,133]. In gliomas, the expression of AURKA is positively correlated with grade I-IV tumors and low patient survival [133,134,135]. Furthermore, AURKA blockade improves the cytotoxic effects of TMZ and ionizing radiation on glioblastoma cells and xenograft models [134,135,136,137]. AURKA has been consistently established as a therapeutic target for glioblastoma. Intriguingly, inhibitors (alisertib, MLN8237) were demonstrated to have restricted efficacy as monotherapy in orthotopic GBM model systems [123,138,139].

Burgess et al.0 obtained an anti-AURKA vNAR (called D01) from a synthetic library established on an isolated scaffold from the Wobbegong shark (*Orectolobus maculatus*), which hampered AURKA through allosteric inhibition mediated by TPX2 (termed vNAR-D01) [122]. AURKA initiation relies on Thr288 autophosphorylation within the activation loop, which is a flexible region [122,140]. Since AURKA autophosphorylation is inefficient, it relies on the microtubule-associated protein TPX2 binding at two different sites in AURKA, which stabilizes αC-helix sites and activation loops through a functional conformational change mechanism analogous to that found in AGC-family kinases such as PKA (Figure 5) [122,141]. The single-domain vNAR-D01 hindered AURKA in a dose-dependent manner. This domain was confirmed to be a competitive inhibitor of TPX2 (Figure 5). vNAR-D01 binding to AURKA was validated through Western blotting. The binding occurred independent of AURKA phosphorylation status. Additionally, vNAR-D01 was bound to the αC-helix, β4 strand, activation loop, and N-terminus of the helix αE regions. Correspondingly, vNAR-D01 variable regions interacted with the kinase surface, as follows: The CDR1 residue Asp33 formed a salt bridge with Arg179 (AURKA αC); HV2 residues 48–49 bound the αE N-terminus; HV2 residues Ser51 and Ile52 bound to the activation loop sequence Val279-His280-Ala281; and CDR3 side chain residues Ile87 and Trp91 attached into a hydrophobic pocket formed between αC and β4 [122]. TPX2 binding preserved the active conformation of AURKA, while vNAR-D01 binding sustained an inactive conformation (Figure 5). Thus, vNAR-D01-specific binding to the AURKA catalytic domain hampers kinase activity via an allosteric mechanism [122]. Although vNAR-D01′s binding affinity (2 µM) requires affinity maturation to improve K_D_ close to at least 100 nM [122], these findings demonstrated the versatility of vNAR. After affinity maturation, these could be employed as intrabodies to target and regulate intracellular kinase mechanisms.

### 4.2. vNAR Domain for the Detection and Intracellular Localization of O-GlcNAc Transferase

**Figure 6:**
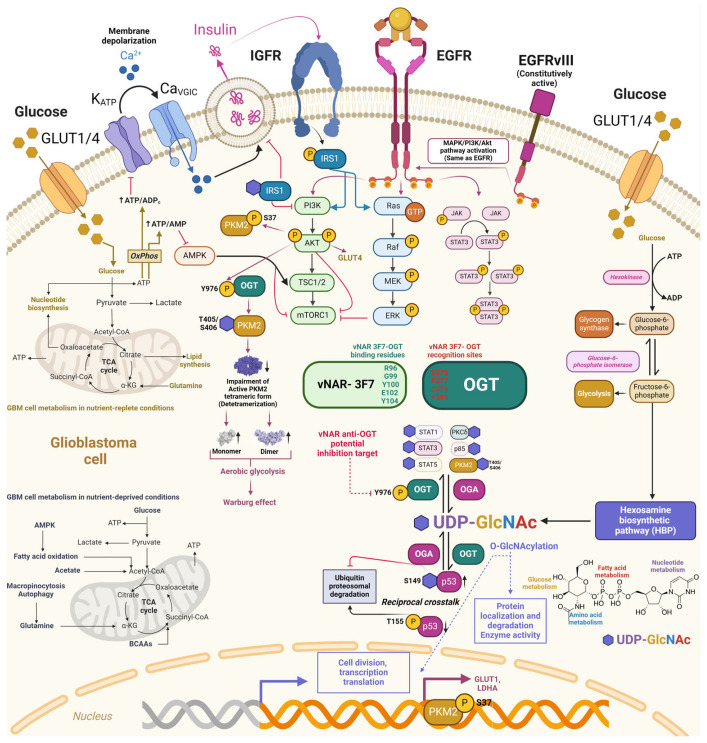
Potential downstream regulation of vNAR-3F7 intrabody through binding to O-GlcNac transferase. O-GlcNAcylation crosstalks with other PTMs, with phosphorylation in serine/threonine residue modifications. Phosphorylation of the tyrosine residue in insulin receptor substrate 1 (IRS1) prompts its association with PI3K and promotes Akt phosphorylation and activation. IRS1 is consequently O-GlcNAcylated to insulin stimulus, hampering IRS1 interaction with PI3K and further decreasing insulin signaling. Deregulated EGF signaling and metabolic shift aerobic glycolysis (Warburg effect) through T405/S406 O-GlcNAcylation of pyruvate kinase M2 (PKM2) mediated by O-GlcNAc transferase (OGT) phosphorylation at the Y976 site further impairs the active PKM2 tetrameric form, increasing PKM2 monomeric and dimeric forms. OGT phosphorylation (Y976) also prompts the O-GlcNAcylation of downstream targets. Anti-OGT vNAR-3F7 recognizes (green) and binds to OGT (red) through specific amino acid residues [160] Copyright © 2023 Xi, Xiao, An, Liu, Liu, Hao, Wang, Song, Yu and Gu. This approach may pave the way for developing anti-OGT vNAR intrabodies that impede intracellular OGT phosphorylation (Y976) and could hamper their corresponding downstream effectors, including PKM2. Dark pink arrows depict EGFR/EGFvIII and downstream targets, whereas blue arrows depict the same for EGFR.

O-N-acetylglucosamine (O-GlcNAc) is a monosaccharose involved in posttranslational modification (PTM) through the covalent addition of serine/threonine residues called O-GlcNAcylation [142]. The insertion and disposal of the O-GlcNAc moiety occur within the cytoplasm, mitochondria, and nucleus, managed solely by a dyad of enzymes, O-β-N-acetylglucosamine transferase (OGT) and O-β-N-acetylglucosamidase (OGA), respectively (Figure 6). O-GlcNAcylation is present in all metazoans, some bacteria, and eukaryotes [142,143,144].

O-GlcNAcylation participates in various biological functions, including protein localization and removal, enzyme activity, transcription, translation, and cellular division (Figure 6) [142]. O-GlcNAcylation has crosstalk with other PTMs, mostly with phosphorylation, since both involve serine/threonine residue modifications, thus potentially competing for identical or adjacent sites. This restrictive regulation is a type of reciprocal crosstalk (Figure 6) [142]. O-GlcNAcylation can inhibit or promote the addition of succeeding PTMs. Additionally, p53 O-GlcNAcylation (Ser149) impedes p53 phosphorylation (Thr155), preventing its ubiquitin–proteasomal degradation. Thus, p53 can enhance apoptotic activity due to its cytoplasmic accumulation (Figure 6) [142,145]. Cellular glucose is regularly consumed in glycolytic pathways. However, 2–5% is employed in the hexosamine biosynthetic pathway (HBP), combining the metabolism of glucose, amino acids, fatty acids, and nucleotides to obtain alpha uridine diphosphate-N-acetylglucosamine (UDP-GlcNAc) (Figure 6) [142], which acts as a nutrient sensor in the cell. O-GlcNAc presents a direct link between the nutrient levels within the cell as a fundamental nutrient sensor and the regulation of relevant biological pathways as a substrate for O-GlcNAcylation [142]. Accordingly, abnormal O-GlcNAc levels were correlated with the pathophysiology of various diseases, including diabetes and cancer [146,147,148]. Aberrant overexpression of insulin growth factor (IGF) and insulin receptors (IGFR) was reported in human cancers [149]. Insulin receptor substrate proteins (IRS) function as the major anchoring proteins of IGFRs [150]. Phosphorylation of the tyrosine residue in insulin receptor substrate 1 (IRS1) prompts IRS1’s association with PI3K and promotes Akt phosphorylation and activation [142,151]. In contrast, IRS1 O-GlcNAcylation decreases IRS1–PI3K interactions, followed by reduced insulin signaling (Figure 6) [142,145,152,153].

Wang et al., (2022) demonstrated a direct connection between EGF signaling deregulation and metabolic shifting toward aerobic glycolysis through the T405/S406 O-GlcNAcylation of pyruvate kinase M2 (PKM2) in breast cancer MCF-7 cells, non-small-cell lung cancer A549, and glioblastoma U251 cells [154]. Stimulation of EGFR can activate tyrosine kinase and PI3K/Akt signaling, prompting the phosphorylation of various crucial enzymes involved in glycolysis (such as pyruvate kinase, phosphofructokinase, and hexokinase) [154,155]. Thus, both enzymes lead to increased glucose uptake while reprogramming metabolism toward backing macromolecular synthesis and NADPH production [154,155]. PKM2 is the gatekeeper enzyme regulating the ultimate phase of glycolysis and plays a key role in the Warburg effect of cancer cells (Figure 6). Thus, the ablation of PKM2 activity mediated by EGF is crucial, especially in cells overexpressing EGFR [154,156,157,158]. In GBM, EGF-mediated activation promoted PKM2 S37 phosphorylation, decreased PKM2 activity, and induced PKM2 nuclear translocation to support lactate dehydrogenase (LDHA) and glucose transporter 1 (GLUT1) expression (Figure 6) [154,158]. Further elucidation of the EGF-mediated PKM2 O-GlcNAcylation mechanism in A549 cells showed that EGF provoked OGT targeting PKM2 by promoting the Y976 tyrosine phosphorylation of OGT. Subsequently, the O-GlcNAcylation of PKM2 was increased, destabilizing the active PKM2 tetrameric form, followed by a significant decrease in PKM2 activity [154]. Upon the O-GlcNAcylation of PKM2, enhanced by EGF, the balance of PKM2 tetramers changed toward dimers and monomers (Figure 6). In addition, overexpression of the EGFRvIII mutant (constitutively active) augmented OGT’s binding to PKM2. EGF-mediated O-GlcNAcylation of PKM2 and detetramerization may impact cell proliferation by regulating PKM2’s metabolic and nuclear functions [154,159]. Interestingly, OGT Y976 phosphorylation also led to increased binding to other phosphotyrosine-binding proteins such as STAT1, STAT3, STAT5, PKCδ, and p85, which were also previously demonstrated to be O-GlcNAcylated (Figure 6) [154]. These discoveries established a direct link between EGF signaling and the O-GlcNAcylation of PKM2, which further decreased PKM2 and led to metabolic reprogramming.

Xi et al., obtained three vNARs against OGT proteins (2D9, 3F7, and 4G2) from an immune phage display-derived vNAR library [160]. The affinity determination of these three anti-OGT vNARs to their corresponding OGT antigens was assessed through ELISA and plasmon resonance [160]. According to the authors, the most reactive, sensitive, and reproducible was vNAR 3F7, which recognized the amino acid residues of the OGT protein at the Ser375, Phe377, Cys379, and Tyr 380 sites through its binding residues Arg96, Gly99, Tyr100, Glu102, and Tyr104 (Figure 6). The affinity of anti-OGT vNAR 3F7 was 53.4 nM [160]. To evaluate the in vitro detection and intracellular delivery of anti-OGT vNAR in NCI-H1299 cells, it was biotinylated for evaluation and OGT localization with ELISA, flow cytometry, and immunofluorescence. The half maximal effective concentration (EC_50_) value for vNAR 3F7 was 40.75 nM. One key drawback to studying O-GlcNAcylation and OGT is the absence of more precise and advantageous research tools [160]. Therefore, the vNAR single domain targeting OGT represents a suitable mechanism for in vitro OGT detection and colocalization to study O-GlcNAcylation and OGT regulatory mechanisms. Moreover, anti-OGT vNARs, including 3F7, could be developed for intracellular expression as intrabodies. As previously mentioned, O-GlcNAcylation is implicated in several examples of cross-talk within the cell. Based on these findings, we also proposed a potential intracellular inhibition target for an anti-OGT vNAR through the blocking of Y976 in O-GlcNac transferase (Figure 6).

### 4.3. vNAR Domain for the Therapeutic Neutralization of TGF-β

**Figure 7:**
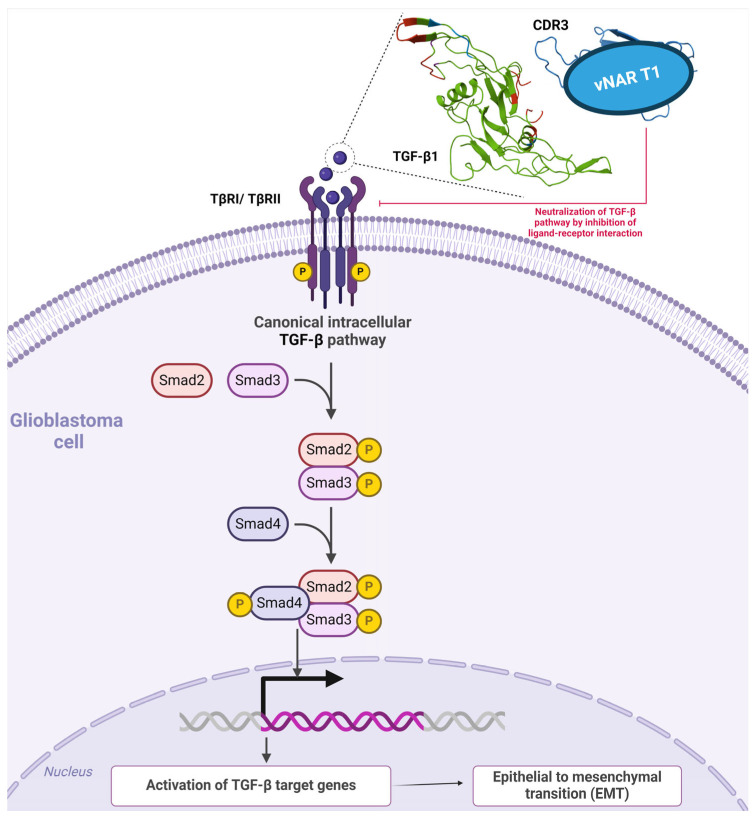
TGF-β canonical intracellular pathways and single-domain vNAR T1 coupled to TGF-β1. In the canonical pathway, TGF-β1 employs two serine/threonine kinase receptors to trigger messenger proteins (SMADs), followed by the activation of TGF-β target genes, which is essential to the onset and progression of GBM. As depicted in the example, TGF-β1 (green) is neutralized by recognition of vNAR T1 (blue) from [172], further hampering TGF-β1 binding to TβI/TβRII receptors. The amino acid sequence of vNAR T1 that recognizes the human TGF-β1 cytokine is depicted in red.

Transforming growth factor-beta (TGF-β) is a cytokine with three isoforms in humans and employs two serine/threonine kinase receptors to trigger messenger proteins (SMADs), thereby inducing the expression of genes essential to multiple roles in the onset and progression of GBM (Figure 7) [161,162]. TGF-β plays a fundamental role in advanced tumors by promoting suppression of immune system responses, tumor proliferation, and metastasis [163,164]. Overexpression of TGF-β and related receptors has been reported in GBM, followed by the development and invasion of TGF-β via TGF-β signaling [165,166,167]. Increased plasma levels of TGF-β were found in GBM patients and diminished following surgical tumor resection [168]. Furthermore, decreased PFS and OS correlate with elevated concentrations of the substrate of TGF-β receptor I (TβRI), phosphorylated SMAD2 (p-SMAD2), in contrast to the concentrations in glioma patients with lower levels [164,169]. Previous studies showed the overexpression of both TGF-β receptors and protein in malignant gliomas, with the mRNA expression of TGF-β1 and TGF-β2 associated with tumor grade [162,164,165,170]. Excessive TGF-β signaling was consistently correlated with a poor prognosis in high-grade gliomas [169]. Using Kaplan–Meier and multivariate analyses, Roy et al., determined a correlation between TGF-β isoform expression and OS and PFS. Interestingly, TGF-β1 played a prevalent role only in newly diagnosed GBM, with 33-fold expression (threefold that of TGF-β2) compared to that in nontumoral samples [162]. Moreover, the authors concluded that moderate to high TGF-β1 correlated with markedly worse OS and PFS in newly diagnosed GBM patients, in contrast to the results with the TGF-β2 isoform. Furthermore, based on a correlation analysis, TGF-β concentrations and target gene expression presented a compelling increase in TGF-β pathway signaling [162]. Moreover, radiotherapy was correlated with increasing TGF-β1 expression levels in vitro and in vivo. Therefore, TGF-β may be most relevant at the first onset of malignant glial tumor development, with decreasing levels after treatment [162]. Since TGF-β isoforms have similar amino acid sequences and engage in intracellular signaling through binding the same receptors and similar subsequent downstream activities, such isoforms remain cell-specific and reliant on expression development [162,171].

Burciaga-Flores et al., isolated the first panspecific single-domain vNAR (vNAR T1) capable of targeting hTGF-β isoforms (β1, β2, and β3) from a nonimmunized *Heterodontus francisci* shark library selected via phage display [172]. The authors then demonstrated the in silico recognition of three TGF-β isoforms under direct ELISA [172]. Based on molecular dynamics, vNAR T1 presented a remarkable CDR3 length comprising 24 amino acid residues, potentially affording more interaction sites and the capability to bind amino acids in the three TGF-β isoforms through the CD3 and HV2 regions [172]. Moreover, vNAR T1 demonstrated a binding preference towards TGF-β1 as the most prevalent isoform in mammals with an affinity (K_D_) of 9.61 × 10^−8^ M [172,173]. vNAR T1 recognizes TGF-β1 amino acid residues (Ile51, Gln57, Lys60, and Arg94), which are essential for binding and interacting with TβRI and TβRII surface receptors [172,174]. Furthermore, vNAR T1 binding occurs within an identical region in the three isoforms, resembling the sequence, function, and binding patterns for the same receptors (Figure 7) [175]. Interestingly, these amino acid residues recognized by vNAR T1 (75% for TβRI and 80% for TβRII), enclosed by vNAR T1’s HV2 and FR1 regions, are also naturally recognized by TGF-β native receptors [174]. These findings further suggest that vNAR T1 is capable of targeting and neutralizing the active form of TGF-β through receptor binding blockade (Figure 7), thereby hindering the corresponding association between TβRI and TβRII and subsequently impeding intracellular TGF-β signaling caused by a failure to assemble the required TβRI2-TβRII2 heterotetramer [171,176,177,178]. Fresolimumab (GC1008) is an mAb able to neutralize all mammalian TGF-β isoforms [179] and is also capable of binding to identical amino acids in TGF-β, such as TβRI and TβRII [164,180]. Nevertheless, mAb tissue penetration is limited due to its considerable molecular weight (~150 kDa) [181]. Due to its low molecular weight (~15–16 kDa), vNAR T1 represents a relevant and advantageous targeted therapeutic strategy. vNAR T1 efficiently binds to excessive TGF-β levels in tissue microenvironments such as those in cancer. Adverse effects caused by the complete neutralization of TGF-β pleiotropic functions within normal tissues are avoided through rapid vNAR T1 clearance via glomerular filtration, which may reduce this potential effect [41,172].

## 5. Conclusions and Future Perspectives

Currently, multimodal therapies have shown limited improvements in glioblastoma treatment, translating into disappointing patient outcomes. Therefore, new targeted therapeutic strategies are still necessary. In this study, we thoroughly summarized the use of single-domain features as intrabodies for cancer and via RME internalization mechanisms in GBM.

We additionally reviewed previously reported vNARs targeting intracellular proteins or receptors also found in GBM. However, these vNARs have not achieved application in targeted glioblastoma therapy. Nevertheless, these vNARs should be seriously considered for this purpose since they target crucial receptors and intracellular proteins relevant to GBM signaling pathways. Among these fascinating vNARs, we also included vNAR-D01 for the allosteric inhibition of Aurora-A kinase, vNARs (2D9, 3F7, and 4G2) for the detection and intracellular localization of O-GlcNAc transferase, and vNAR T1 for the therapeutic neutralization of TGF-β. In the context of GBM, targeted O-GlcNAc transferase (OGT) could also be relevant for the Y976 tyrosine phosphorylation site by blocking metabolic reprogramming via PKM2 or binding to downstream proteins relevant to GBM pathogenesis.

During the writing of this paper, a study on vNARs (described as clone 3 and clone 5) was published, presenting vNARs as intrabodies for CTS enzyme neutralization that act by diminishing cancer cell proliferation. This article demonstrated that vNARs could be relevant sdAbs for intrabody applications such as VHH. Therefore, vNARs are an excellent option for developing new targeted intracellular therapy, with the possibility of humanization, solubilization improvement, and in silico affinity maturation. All the positive characteristics of vNARs suggest the need to create biotechnology-based companies worldwide to accelerate the transition from the laboratory to preclinical or clinical trials for new intrabodies against cancer.

Furthermore, vNARs could provide beneficial results for GBM in the targeted inhibition of signaling pathways by employing intrabodies or RME within tumor cells. We examined the potential of using these reported vNARs in the specific inhibition of intracellular targets to neutralize or modulate the relevant pathways involved in the onset and development of glioblastoma for potential therapeutics based on the heavy domains themselves or the carrying of antitumor drugs.

## Figures and Tables

**Table 3:** Summary of SdAbs against GBM.

SdAb	Source	Target	Internalization Mechanism	Strategy	Results	Ref.
EG2-Cys	Camelid	EGFRvIII	Receptor-mediated endocytosis (RME) via EGFRvIII binding	Near-infrared quantum dot (Qd800) conjugation to an anti-EGFRvIII nanobody for cell imaging	EG2-Cys was internalized in vitro and in vivo (orthotopic GBM mouse model) in EGFRvIII-positive glioblastoma cells (U87MG).	[107]
VUN100	Camelid	US28	US28 binding and constitutive endocytosis of chemokine receptor US28	Photosensitizer IRDye700DX conjugation to an anti-US28 nanobody for targete55d PDT	VUN100-PS conjugate induced cell toxicity of US28-expressing GBM cells (U251) in 2D and 3D cultures after 1 h of incubation in vitro.	[109]
13R_VNAR_102 13R_VNAR_106	Shark	IL-13Rα2	Receptor-mediated endocytosis (RME) via IL-13Rα2 binding	Isolation, characterization, and evaluation of two anti- IL-13Rα2 nanobodies incubation with three concentration gradients	Incubation with these vNARs showed strong inhibitory ability on the growth and migration of IL-13Rα2 highly expressed GBM cells (A172) in vitro.	[111]
Nb79	Camelid	VIM	Not specified	Consecutive treatment with Nb79 (anti-VIM) and Nb225 (anti-TUFM)	Nb79 reduced survival of GBM cells (U87MG, >50%; U251MG cells, 40%). Further, reduced stem cell line survival (NCH421k, >50%; NCH644 cells ~80%).	[112]
Nb225	Camelid	TUFM	Not specified	Consecutive treatments with Nb79, Nb179 and Nb312	Nb79 and Nb225: The survival of U251MG and U87MG cells was reduced (76% and 50%, respectively). NB179 and Nb225: Decreased the survival of U87MG cells significantly (46%), and that of NCH644 cells (45%). Nb225 and Nb314: Significantly reduced survival of U251MG (30%), U87MG (32%) and NCH644 cells (42%).	
Nb179	Camelid	NAP1L1	Not specified	Consecutive treatment with Nb179 (anti-NAP1L1) and Nb225 (anti-TUFM)	Nb179 and Nb225 decreased the survival of GBM cells U87MG, (46%) and GBM stem cells NCH644 cells (45%).	
Nb314	Camelid	DPSYL2	Not specified	Consecutive treatment with Nb134 (anti-DPSYL2) and Nb225 (anti-TUFM)	Nb225 and Nb314 significantly reduced survival of GBM cells (U251MG, (30%). U87MG (32%) and GBM stem cells NCH644 (42%).	

GBM: glioblastoma; RME: receptor-mediated endocytosis; EGFRvIII: the epidermal growth factor receptor variant III; Qd: quantum dots; PDT: photodynamic therapy; vimentin (VIM); TUFM: mitochondrial protein Tu translation elongation factor; NAP1L1: nucleosome assembly protein 1-like 1; DPSYL2: dihydropyrimidinase-related protein 2.

## Data Availability

No new data were created or analyzed in this study. Data sharing is not applicable to this article.
